# Reliable and accurate data capture using tablets, phones or other mobile devices

**DOI:** 10.1186/1745-6215-16-S2-P29

**Published:** 2015-11-16

**Authors:** Mihaela Barbu

**Affiliations:** 1 Swansea University, Swansea, UK

## 

We collect, randomise, exploit and visualise clinical research data on mobile platforms; for this reason, you really need to know what the data looks and feels like, based on database specification. A common challenge is how to store and present informative meta data to researchers.

Working with our colleagues from Swansea University, we have developed an online randomisation system to be accessed from mobiles, in order to obtain an instantaneous allocation result for a Social Work Intervention trial.

We will describe the process and technology involved in developing this system. We will present the mobile platform section, the documentation that enables researchers to capture and share the methodology used to create study variables. This successful implementation of the online application represents a step forward in enabling researchers to decide how the data can be used for their research and also offers the opportunity for researchers to share conceptual methodologies.

